# An Exposure Assessment of Arsenic and Other Trace Elements in Ha Nam Province, Northern Vietnam

**DOI:** 10.1155/2019/5037532

**Published:** 2019-12-21

**Authors:** Manh Ha Nguyen, Quoc Anh Hoang, Lan Anh Nguyen, Thi Thao Ta, Tien Duc Pham, Minh Binh Tu, Dinh Binh Chu

**Affiliations:** ^1^Faculty of Chemistry, VNU University of Science, Vietnam National University, 19 Le Thanh Tong, Hoan Kiem, Hanoi 100000, Vietnam; ^2^School of Chemical Engineering, Hanoi University of Science and Technology, 1 Dai Co Viet, Hai Ba Trung, Hanoi 100000, Vietnam

## Abstract

Concentrations of As and other trace elements were measured in groundwater, rice, hair, urine, and blood samples of people consuming As-contaminated groundwater in a village of Ha Nam province, northern Vietnam to understand the recent status of contamination and assess the possible risks of human exposure. Elevated concentrations of As in groundwater were still observed, exceeding the WHO guideline value in most of the tube wells investigated. Significant positive correlations between As concentrations in groundwater and human samples (hair and urine) were observed. Arsenic concentrations in human and hair appeared to be related to the groundwater usage habit, with higher levels found in drinking group than those in the washing group. Significant good correlations were also encountered between cumulative intakes of As, Mn, and Ba through groundwater consumption and hair concentrations. All these results indicate the chronic exposure to As and some other elements such as Mn and Ba. The total intakes of As, Mn, and Ba through rice and groundwater consumption were estimated to be ranged from 80–836, 49.3–1850, and 311–97100 *µ*g/day, respectively. The daily intakes of As of the study area ranged from 1.6–16.7 *µ*g/kg body wt./day, mean: 7.15 *µ*g/kg body wt./day, in which about 85% of the subjects were above the provisional tolerable daily intake proposed by WHO.

## 1. Introduction

In recent years, natural contamination by arsenic in groundwater has received considerable attention in many countries around the world such as India, Bangladesh, Chile, and Taiwan [1–4]. In Vietnam, the issue of arsenic contamination in groundwater and drinking water was discovered in 2001, in which elevated As concentrations were found in various areas in the Red River Delta, northern Vietnam [5]. Human exposure studies have been continuously conducted, and results indicated widespread contamination in both the Red River Delta in the north and Mekong River Delta in the south of Vietnam (review by Agusa et al. [6]). Chronic exposure to arsenic from As-contaminated groundwater has been suggested as main sources of human exposure. Severe arsenic poisoning symptom in Vietnam has also been reported [7, 8].

Most of the studies on human exposure to Arsenic in Vietnam have focused on the measurement of As in human hair and urine and very limited information on the levels of As in blood serum samples [6]. While many studies have been conducted, a comprehensive data set on the levels of As and other trace elements in paired tube-well water, human samples, and food sources in As-contaminated areas in Vietnam is still very limited. Such information is necessary for an in-depth evaluation of the extent of As contamination and risk for As exposure through drinking water and other intake sources.

In this study, we conducted a survey to collect groundwater from tube-wells of families in a village of Ha Nam province, northern Vietnam, where high and widespread contamination of As has recently been reported [9]. In addition, human samples (hair, urine, and serum) and rice samples from these families were also collected and concentrations of As and other trace elements were measured in order to provide a comprehensive assessment of the recent contamination status, human exposure, and risk assessment based on the intake doses of As consumption through rice and contaminated groundwater.

## 2. Materials and Methods

### 2.1. Sample Collection and Storage

Groundwater samples were randomly collected from 21 families in Nhat Tan villages, Ha Nam province, northern Vietnam during September 2018. Information of groundwater and human samples is given in Table 1. In each family, unfiltered and sand filtered, or rainy water samples were simultaneously collected. Polypropylene bottles (100 mL), which were washed with Milli-Q water, were used for water sampling. The collected groundwater samples were acidified (pH < 2) with extrapure HCl for As and HNO_3_ for other elements and stored at −5°C as immediately as possible.

Human samples including urine, hair, and serum samples were also collected from the same families with groundwater collection. Our questionnaire survey indicates that there are two main groups of human samples, according to the habit of groundwater usage. One group uses groundwater for drinking, and the other uses it mainly for washing. Food consumption items of the people in the surveyed village are mainly rice, vegetables, egg, and pork. Spot urine samples were collected in a plastic tube (Tube Traite, 50 mL, NJ, USA) and then stored at 4°C for storage and transported to the laboratory and analyzed immediately. Blood samples were taken with disposable needles inserted into the plastic tubes (Tube Traite, NJ, USA) after clotting the sera. After that, the collected sera samples were kept at 4°C and then transported to the laboratory immediately within a day; other sera samples were stored at −18°C. Hair and rice samples were kept in a zipped plastic bag and stored in a dry chamber at 20°C until analysis. Prior to the analysis, all rice samples were transferred into open, precleaned, and preweighed plastic tubes. Informed consent of all donors for using human samples for environmental exposure research was obtained.

### 2.2. Chemical Analyses

All single-element stock standard solutions (1000 *µ*g·mL^−1^) in HNO_3_ or HCl for ICP-MS or AAS were purchased from Merck (Singapore). Multielement working standard solution was prepared weekly by dilution of the stock solution in 5% HNO_3_ in ultrapure water and kept at 4°C in an amber HDPE bottle.

For multielemental analysis: an ELAN 9000 ICP-MS (PerkinElmer Sciex, Penlivia Canada) system including a liquid autosampler was used for multielemental analysis. Sensitivity and performance of the ICP-MS instrument was daily checked by using tuning solution (Perkin Elmer).

### 2.3. Sample Preparation

#### 2.3.1. Water Samples

After transporting to the laboratory, water samples were acidified with H_2_SO_4_ for As and HNO_3_ for other elements. Milli-Q water acidified with H_2_SO_4_ or HNO_3_ was used as control. Arsenic concentration in water samples was measured by hydride generation atomic absorption spectrometry (AAS) using a Shimadzu HVG-1 hydride system coupled to a Shimadzu-AA680 AAS [10]. Concentrations of Cu, Pb, Cd, Zn, Co, B, Se, Mo, Mn, Sb, Cr, Ba, and Fe were determined by inductively coupled plasma mass spectrometry (ICP-MS; Hewlett–Packard, HP-4500) [11]. Indium was used as an internal standard for ICP-MS measurements. Water pH was measured by a glass electrode pH meter (Asahi Techno Glass).

#### 2.3.2. Rice Samples

An approximately 0.2 g freeze-dried rice sample was weighed into a 75 mL Teflon microwave digestion vessel, and 6 mL of concentrated nitric acid was added and stood overnight. Afterward, rice sample was digested in a microwave oven (Multiwave PRO 50 HZ Package 24HVT8, Anton Paar, Graz, Austria). The temperature program was increased up to 165°C in 15 min and then held at this temperature for a further 10 min. In the second step of the digestion, the vessels were cooled to 50^o^C for 20 minutes. After digestion, the samples were cooled to room temperature and transferred quantitatively into 50 mL volumetric flasks and made up to volume with DIW. Solutions were analyzed for the total arsenic content by inductively coupled plasma-dynamic reaction cell quadrupole mass spectrometry (ICP-DRC-QMS) employing with oxygen gas in order to eliminate polyatomic interferences. For quality control and method validation, rice-based certificated reference material (ERM BC-211) was prepared and analyzed at the same time.

#### 2.3.3. Serum and Urine Samples

The sample treatment procedure for As and other trace elements in urine, serum, and hair samples followed the method described in our previous study [12]. Urine and serum samples were digested in an acidic condition in a microwave oven for total As determination. In brief, 0.1 mL of urine or serum was poured into a microwave cell, and 2 mL concentrated nitric acid (Suprapure, Merck, Singapore) and 1 mL H_2_O_2_ (Merck, Singapore) were added, followed by digestion at 80% power of an Anton PARR (Graz, Austria) microwave oven. After digestion, the clear solution was transferred to a precleaned 15 mL polypropylene tube and deionized water was added to 5 ml. These solutions were subjected to analysis by ICP-MS using indium as the internal standard. Fish-based matrix-certified reference material (DORM III and DORM IV from National Research Council, Canada and BRC 627, BRC 211 from Institute for Reference Materials and Measurements, Belgium) was used for quality control of total arsenic analysis. Practically, the number of quality control and blank samples accounted for 20% of the number of total samples that were subjected to total analysis.

#### 2.3.4. Hair Samples

Human hair samples were washed by sonication with 0.5% surfactant reagent (polyoxyethylene lauryl ether) and subsequently dried for 12 h at 80°CC. For As analysis, a dried hair sample was accurately weighted directly into a Kjeldahl flask and 8 ml of purified HNO_3_ was added. After predigestion at room temperature overnight, the sample was treated with 16 ml of acid mixture (HNO_3_ : HClO_4_ : H_2_SO_4_ 1 : 2 : 1) and digested by heating to over 300°C until the perchloric acid was removed. Arsenic concentration was measured by HG-AAS. For analysis of other trace elements, about 0.1 g of the hair sample was digested in 1.5 ml of concentrated HNO_3_ in a Teflon PTFE tube in a microwave oven with the same manner as with rice and serum and urine samples. Concentrations of 13 elements (Cu, Pb, Cd, Zn, Co, B, Se, Mo, Mn, Sb, Cr, Ba, and Fe) were determined by ICP-MS. The accuracy of the method was assessed using spiking experiments. Recoveries of the elements ranged from 80 to 118%.

### 2.4. Statistical Analysis

Statistical analysis was performed using Microsoft Excel (Microsoft Office 2010) and Minitab 16® Statistical Software (Minitab Inc.). The whole data set were analyzed by Pearson correlation analysis to find out possible relationships between the target compounds. The level of statistical significance was set at *p* < 0.05.

## 3. Results and Discussion

### 3.1. Contamination of Arsenic and Other Trace Elements in Groundwater and Rice

Concentrations of arsenic in groundwater collected from tube wells of 20 families ranged from <0.01 to 467 *µ*g/l, with mean and median concentrations of 109 and 89.9 *µ*g/l, respectively (Table 2). Most of the unfiltered groundwater samples (20/21) contained As concentrations exceeding the WHO guideline value of 10 *µ*g/l with the highest As concentration of 467 *µ*g/l [13]. Water samples from the household containers of each family, which were mixed of filtrated groundwater and rain water, were also collected and examined. As concentrations in filtered groundwater and rain water were remarkably reduced as compared with those in untreated groundwater, ranging from 1.12–135 *µ*g/l (mean: 44.2 *µ*g/l).

It should be noted that 70% of filtered water samples (14/21 samples) contained As concentrations exceeding the WHO guideline value. This result indicates that As contamination in Ha Nam province is still relatively serious. As concentrations in Nhat Tan village were higher than those reported in several sites in Ha Nam province (Liem Thuan and Nhan Dao), in some districts of Hanoi city (Gia Lam, Dong Anh, and Tu Liem), but lower than those from Bo De, Hoa Hau, and Vinh Tru in the province [6, 8]. As concentrations in Nhat Tan were still lower than those in Chuyen Ngoai and Chau Giang village of Ha Nam province [9]. Many of the sand-filtered groundwater samples still contained elevated As concentrations. Our data is consistent with that from a previous study, suggesting that a household sand filtration system is not efficient enough to reduce As contamination to the safe level [9].

Concentrations of other elements in groundwater are given in Table 2. Concentrations of Fe, Ba, Mo, and B were higher than other elements. Meanwhile, toxic heavy metals such as Pb, Zn, and Cd showed relatively low contamination levels. Mean concentrations of Sb, Mn, and Ba in groundwater from Nhat Tan were 25.7, 23.8, and 2380 *µ*g/l, respectively. About 50% of groundwater samples contained Sb concentrations exceeding the WHO drinking water guideline value of 5 *µ*g/l. As for Ba, concentrations ranged from 0.47–91600 *µ*g/l (mean: 2380 *µ*g/l), with two samples having elevated concentrations exceeding the WHO guideline value (700 *µ*g/l). Mn concentrations were from <0.01 to 257 *µ*g/l, and no sample had concentrations beyond the WHO guideline level. Our previous study from other villages in Ha Nam province also showed similar contamination levels and distribution, with elevated concentrations of As and Ba with significant numbers of samples having concentrations exceeding the WHO guideline value [9]. This fact suggests widespread contamination of As and other trace elements such as Mn and Ba from Ha Nam province.

Concentrations of As in rice collected from each family along with groundwater ranged from 0.05 to 0.33 *µ*g/g, mean: 0.09 *µ*g/g. Fe, Zn, and Mo showed higher concentrations than other elements. Cu, Pb, and Ba were at moderate levels, ranging from 4.9 to 5.5 *µ*g/g. In Ha nam Province, As concentrations in rice from Nhat Tan were lower higher than those reported in Vinh Tru, Ha Tay, Hanoi [6], and some locations in the Red River Delta [14].

### 3.2. Contamination by As and Other Elements in Human Samples

Concentrations of As in hair and urine of people of 21 families consuming groundwater ranged from 0.1 to 2.5 *µ*g/g (mean: 0.59 *µ*g/g) and 0.7 to 28.1 (mean: 5.12 *µ*g/g creatinine), respectively (Table 2). As concentrations in groundwater significantly correlated with those in both hair and urine of male samples (*r* = 0.63; *p* < 0.05). Similar significant correlations were observed between groundwater and hair of human-consumed As-contaminated water from suburban areas of Hanoi and 4 villages of Ha Nam province [8, 15]. This result continues to support long-term chronic exposure to As through consumption of contaminated groundwater. The As levels in hair and urine were generally in the range to those reported in Hoa Hau, Liem Thuan, Bo De, Chuyen Ngoai, and Chau Giang villages in Ha Nam province [6, 8, 9]. Our data in Nhat Tan village further indicate widespread groundwater contamination and chronic human exposure to As in Ha Nam province.

**Figure 1:**
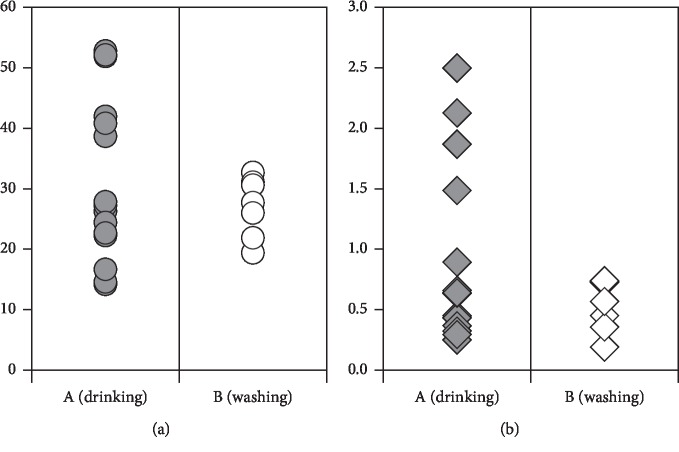
Concentrations of As in urine and hair samples of two groups using groundwater for drinking (A) and washing (B). Exposure levels of group A were significantly higher than those of group B (Mann–Whitney *U* test, *p* < 0.05). (a) As concentration in urine (*μ*g/l). (b) As concentration in hair (*μ*g/g).

To evaluate the possible factors influencing As contamination in human samples, As exposure in hair and urine according to the groundwater usage was examined (Figure 1). An interesting result was observed, showing higher degree of contamination in hair and urine of people using groundwater for drinking as compared with washing. Unlike other locations such as Bangladesh, the groundwater usage habit in Vietnam varied among locations and families. Our questionnaire survey indicated that there are two main groups: one using water for drinking and washing and the other mainly using groundwater for washing and rain water for drinking. Higher As concentrations in hair and urine samples of the drinking group clearly suggest that the major pathway of As exposure in the investigated groups is via the contaminated groundwater. In hair samples, there are 4 samples containing As concentrations greater than the level associated with skin disease of 1 *µ*g/g [16].

Among trace elements, Ba, Mo, Sb, and Zn accumulated higher levels than the others (Table 2). Concentrations of Pb and Cd in urine were relatively low. Their residue levels in hair and serum ranged from <0.01 to 132 and < 0.01 to 3.89 *µ*g/g, respectively, while Cu and Zn accumulated higher concentrations than Pb and Cd. As for Ba, despite elevated concentrations in groundwater, concentrations in hair and urine were at moderate levels. Mn concentrations in hair samples were 0.26–34.4 *µ*g/g, mean: 4.47 *µ*g/g. Only one hair sample contained Mn concentrations exceeding the levels associated with chronic Mn poisoning [17, 18]. However, given the elevated concentrations of Mn and Ba in some human samples, the toxic effects of these elements are of concern, and further studies are needed to provide in-depth assessment of relationship between their human exposure and toxic effects.

As concentrations in serum samples ranged from 22.5 to 482 *µ*g/g creatinine (1.09–25.2 *µ*g/l, mean: 11.2 *µ*g/l). There are almost no data available for As in blood samples from groundwater contaminated areas in Vietnam. Our data in Ha Nam province was comparable with those reported in national survey in Bangladesh and slightly lower than the mean blood level of people identified with skin lesion (14.3 *µ*g/l, *n* = 303) [19]. The serum As concentrations in Vietnam were higher than those in both healthy people and patients with various diseases reported in Belgium [20]. As for other elements, mean concentrations of Pb and Cd were from 6.44 to 0.52 *µ*g/l, respectively, which were comparable or higher than those reported for patients with acute hemorrhagic stroke in Turkey [21]. In general, toxic element concentrations in serum from Ha Nam were in the range of those reported in human samples with toxic element-associated diseases. Further studies are therefore needed to evaluate long-term accumulation and relationship between levels of As and other toxic elements from As-contaminated areas in Vietnam.

### 3.3. Exposure Assessment of As and Other Trace Elements

To further evaluate the accumulation and source of exposure of As and trace elements, correlations between various kinds of samples (water, rice, urine, hair, and serum) were examined, and the result of the significant relationships is given in Table 3. Significant correlations between As water and As urine and hair in males were observed. As for rice samples, good correlations were also recorded in male and female hair and female urine. Serum and urine As in males and females showed significant correlations. Levels in urine and hair reflect current and long-term exposure. All these observations suggest the chronic exposure to As through consumption of water and rice. Similar results with significant positive correlations between As concentrations in groundwater and As concentrations in urine and hair from various villages from Ha Nam [6, 8, 20]. In the present study, we provide more comprehensive data with As exposure to different kinds of samples including water and rice and three types of human samples.

To evaluate the risk for cumulative exposure to As, Mn, and Ba through contaminated groundwater, we estimated intakes of these elements based on the age of wells, annual ingestion rate of groundwater, and daily water consumption. The detailed descriptions for calculation of cumulative intakes are described in Agusa et al. [15]. The cumulative intakes (CI) and daily intakes (DI) of As, Mn, and Ba were estimated for residents in the studied area based on the following equations [6, 15]:

(1) $$\begin{array}{c} \text{CI }\left(\mu \text{g}\right)=\text{Concentration of element in groundwater }\left(\mu \text{g}/\text{L}\right)\times \text{Water utilization time }\left(\text{year}\right)\\ \times \text{Annual ingestion rate of groundwater }\left(\text{day}/\text{year}\right)\times \text{Water consumption rate }\left(\text{L}/\text{day}\right),\\ \text{DI }\left(\mu \text{g}/\text{day}\right)=\text{Concentration of element in groundwater }\left(\mu \text{g}/\text{L}\right)\\ \times \text{Water consumption rate }\left(\text{L}/\text{day}\right)+\text{Concentration of element in rice }\left(\mu \text{g}/\text{g}\right)\\ \times \text{Rice consumption rate }\left(\text{g}/\text{day}\right). \end{array}$$

The annual ingestion rate of groundwater was assigned as 182.5 days per year, implying the use of groundwater during dry seasons [15]. The water consumption rate was 2 L/day [13]. A rice consumption rate of 369.1 g/day for the Red River Delta region was retrieved from the General Nutrition Survey from 2009 to 2010 [22]. The daily intake doses (DID, *μ*g/kg/day) of these elements were calculated by the abovementioned DI values with an average body weight of 50 kg and compared with the tolerable daily intakes (TDIs) proposed by the World Health Organization (WHO) [23].

**Figure 2:**
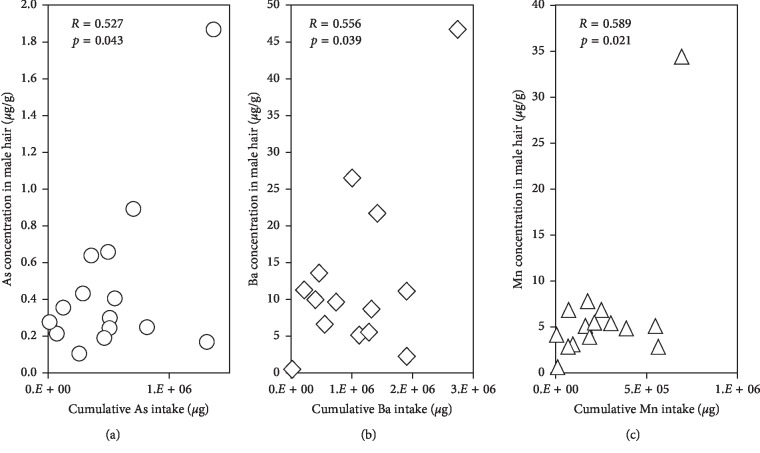
Relationships between the cumulative intakes of (a) As, (b) Ba, and (c) Mn through contaminated groundwater and their concentrations in human hair samples (Pearson correlation coefficients *R* > 0.500, *p* < 0.05).

We found significant positive correlations between cumulative intakes of As, Mn, and Ba and their concentrations in human hair (Figure 2). A similar result was observed in a previous survey in Hanoi suburban areas and some villages in Ha Nam [9, 15]. Our data suggest that people living the investigated area in Ha Nam province are chronically exposed to As, Mn, and Ba through consumption of contaminated groundwater.

In addition to exposure through groundwater, intakes through rice is considered to be an important pathway because rice is one of the major foods for Vietnamese people. Paired samples of rice and groundwater of investigated families are available for estimation of total intakes of trace elements. Means and ranges of total intake of As, Mn, and Ba through rice and groundwater consumption are given in Table 4. The detailed description of estimation of total intakes is described in Agusa et al. [6]. The total intakes of As, Mn, and Ba were in the range of 80–836, 49.3–1850, and 311–97110 *µ*g/day, respectively. In particular, the mean As intake was 357 *µ*g/day, which was lower than those previously estimated in As-contaminated sites in Vietnam, but comparable or higher than those in some places in Bangladesh [20, 24].

However, the As intakes were apparently higher than those in noncontaminated sites [25, 26]. The intake values of As estimated per 1 kilogram of body weight were ranged from 1.6–16.7 *µ*g/kg·body wt./day, mean: 7.15 *µ*g/kg·body wt./day. Daily intakes for about 80% the subjects were above the provisional tolerable intake value proposed by the WHO (3 *µ*g/kg·body wt./day) [23]. This fact raises concern over the elevated chronic exposure to As and suggests the needs for continued comprehensive surveys in a larger geographical area in Vietnam.

Health risks for human were evaluated from values of chronic risk and carcinogenic risk. The value of chronic risks can be calculated by the ratio between the estimated exposure (average daily intake-ADD) and the reference dose (RfD) called the “Hazard Quotient” (HQ).

(2) $$\begin{array}{c} \text{HQ }=\frac{\text{ADD}}{\text{RfD}}. \end{array}$$

The risk is considered when HQs > 1.

Besides, carcinogenic risk can be calculated as follows:

(3) $$\begin{array}{c} \text{R}=1-{\text{e}}^{-\left(\text{SF }\times \text{ADD}\right)}, \end{array}$$

where SF is the slop factor. The detailed description of the risk assessment using HQ and SF is given in our previous study [9].

As the results of high arsenic consumption through the drinking water pathway, both potential chronic and carcinogenic risks of the two groups were calculated. Approximately, 52% of the families using filtered groundwater and 86% of the families using unfiltered groundwater could be affected by arsenic. The value of carcinogenic risk index for residents using unfiltered and filtered groundwater was estimated to be 3 in 1,000 people and 8 in 10,000 people. While the ratio of 1 in 1,000,000 is considered to be significant by the US-EPA, the carcinogenic rate values estimated above suggest high potential risk.

## 4. Conclusion

In summary, the present study continues to indicate elevated and widespread contamination by As in groundwater in Ha Nam province. Residents living in the investigated area are chronically exposed to As, Mn, and Ba. Estimated daily intakes of As were still relatively high, with significant numbers of subjects exceeding the WHO tolerable intake value. Further monitoring on the As speciation in groundwater and human samples and toxicologic studies along with development of effective treatment technologies to reduce As levels in groundwater is urgently needed to mitigate risk for elevated and chronic As exposure.

## Figures and Tables

**Table 1:** Information on groundwater and human samples analyzed in this study.

Sample	Groundwater	Hair	Urine	Blood
Sample number	21 unfiltered water 21 filtered or rain water	35	29	29
Water utilization time (year)	16 (10–26)			
Age (year)		44 (23–70)	44 (23–70)	44 (23–70)

**Table 2:** Concentrations of arsenic and other trace elements in groundwater, rice, and human samples collected from Ha Nam province, Northern Vietnam.

Sample	Cu	Pb	Cd	Zn	Co	B	Se	Mo	Mn	Sb	Cr	As	Ba	Fe
Groundwater (*µ*g/L)														
*n*	42	42	42	42	42	42	42	42	42	42	42	42	42	42
Mean	3.74	0.28	0.13	19.8	0.63	572	1.2	1970	40.8	25.7	5.31	109	2385	3335
Median	<0.01	<0.01	<0.01	<0.1	0.42	503	1.18	1632	27.9	5.2	5.31	89.9	154	495
Range	<0.01–134	<0.01–6.56	<0.01–5.38	<0.01–271	<0.005–4.05	<0.01–2160	<0.02–2.97	149–8560	<0.01–257	<0.1–180	2.62–9.55	1.12–467	0.47–91580	16.8–24770
SD	20.506	1.098	0.819	56.248	0.731	534	0.754	1712	49.8	44.3	1.76	108.9	13930	5343

Rice (*µ*g/g)														
*n*	21	21	21	21	21	21	21	21	21	21	21	21	21	21
Mean	4.93	5.55	0.09	172	0.03	0.84	0.42	82.9	1.68	13.8	0.08	0.09	4.9	21.3
Median	4.31	1.05	0.05	113	0.01	0.22	0.3	54.7	0.99	7.08	0.06	0.08	2.82	16.4
Range	<0.01–18.3	(<0.01–90.7)	(0.05–0.29)	3.20–661	<0.005–0.14	<0.01–9.92	0.07–2.68	<0.01–247	<0.01–4.66	<0.1–58.5	<0.01–0.5	0.05–0.33	0.23–25.0	3.44–102
SD	32.6	19.1	0.09	152	0.04	2.1	0.52	61.6	1.54	16.5	0.12	0.06	5.88	20.4

Urine (*µ*g/g creatinine)														
*n*	29	29	29	29	29	29	29	29	29	29	29	29	29	29
Mean	1.28	<0.01	0.12	34.1	0.05	2625	2.37	25240	0.41	1.58	4.07	5.12	1.06	15.8
Median	0.75	<0.01	0.05	14.1	0.02	1084	1.21	11050	0.03	0.96	2.78	2.74	0.19	6.89
Range	0.07–8.83	<0.01	(<0.01–0.84)	0.50–256	<0.005–0.53	281–14392	<0.02–13.7	2236–227300	<0.01–7.59	<0.1–7.60	0.55–16.8	0.70–28.1	0.03–16.9	2.01–103
SD	1.68	<0.01	0.16	47	0.1	3389	3.54	44580	1.33	1.66	4.01	5.47	2.85	20.5

Hair (*µ*g/g)														
*n*	35	35	35	35	35	35	35	35	35	35	35	35	35	35
Mean	16.40	5.45	0.46	321	0.14	0.03	4.68	93.70	4.47	73.20	0.29	0.59	10.40	47.50
Median	8.48	1.26	0.20	207	0.05	0.01	0.55	76.40	3.89	24.20	0.10	0.42	8.71	37.50
Range	3.46–164	(<0.01–132)	(<0.01–3.89)	104–1134	<0.005–1.44	<0.01–0.43	0.21–125	<0.01–287	0.26–34.4	<0.01–694	<0.01–2.90	0.10–2.50	<0.01–46.7	8.1–144
SD	29.9	21.8	0.8	261.0	0.3	0.1	20.8	67.8	5.7	155.0	0.6	0.5	9.9	29.7

Serum (*µ*g/g creatinine)														
*n*	29	29	29	29	29	29	29	29	29	29	29	29	29	29
Mean	127	108	8.92	3161	2.36	0.43	9.54	1681	15.6	1531	4.95	187	124	2223
Median	103	24.4	4.3	2469	0.69	0.13	8.87	1388	6.16	387	2.19	147	37.3	1861
Range	5.76–282	<0.01–2149	0.41–77.5	239–6395	0.16–18.5	<0.01–5.15	3.40–16.5	<0.01–4445	<0.01–214	<0.1–13724	<0.01–35.3	22.5–482	2.72–1108	434–6958
SD	70.4	387	14.9	2350	4.33	0.99	3.19	1243	39.2	3150	8.36	138	238	1502

**Table 3:** Results of statistical analyses for the correlations between As concentrations in different kinds of samples.

Paired samples	Correlation coefficient *R*	*p* value
Water-male urine	0.605	0.049
Water-male hair	0.626	0.039
Rice-female hair	0.941	0.001
Rice-male hair	0.686	0.02
Rice-female urine	0.755	0.007
Female hair-female urine	0.654	0.029

**Table 4:** Daily intakes (*μ*g/day) of As, Mn, and Ba through consumption of groundwater and rice estimated for residents in Nhat Tan Village, Ha Nam Province.

		Water consumption	Rice consumption	Total daily intakes
As	Mean ± SD	218 ± 130	139 ± 145	357 ± 220
	Range	2.76–471	3.04–578	80.0–836

Mn	Mean ± SD	81.7 ± 62.0	621 ± 581	702 ± 572
	Range	1.92–258	30.0–1720	49.3–1850

Ba	Mean ± SD	4770 ± 20,000	1800 ± 2230	6580 ± 20,800
	Range	4.13–92,000	83.6–9230	311–97,100

## Data Availability

The data used to support the findings of this study are included within the article.

## References

[1] Chowdhury U. K., Biswas B. K., Chowdhury T. R. (2000). Groundwater arsenic contamination in Bangladesh and West Bengal, India. *Environmental Health Perspectives*.

[2] Anawar H. M., Akai J., Mostofa K. M. G., Safiullah S., Tareq S. M. (2002). Arsenic poisoning in groundwater: health risk and geochemical sources in Bangladesh. *Environment International*.

[3] Marshall G., Ferreccio C., Yuan Y. (2007). Fifty-Year study of lung and bladder cancer mortality in Chile related to arsenic in drinking water. *JNCI Journal of the National Cancer Institute*.

[4] Huang Y.-K., Huang Y.-L., Hsueh Y.-M., Wang J. T.-J., Yang M.-H., Chen C.-J. (2009). Changes in urinary arsenic methylation profiles in a 15-year interval after cessation of arsenic ingestion in Southwest Taiwan. *Environmental Health Perspectives*.

[5] Berg M., Tran H. C., Nguyen T. C., Pham H. V., Schertenleib R., Giger W. (2001). Arsenic contamination of groundwater and drinking water in Vietnam: a human health threat. *Environmental Science & Technology*.

[6] Agusa T., Kunito T., Kubota R. (2010). Exposure, metabolism, and health effects of arsenic in residents from arsenic-contaminated groundwater areas of Vietnam and Cambodia: a Review. *Reviews on Environmental Health*.

[7] Dang M. N., Nguyen K. H., Chander B., Nguyen Q. H. The Adverse Effects of Arsenic on Population Health in Selected Communities of Ha Nam Province.

[8] Nguyen V. A., Bang S., Viet P. H., Kim K.-W. (2009). Contamination of groundwater and risk assessment for arsenic exposure in Ha Nam province, Vietnam. *Environment International*.

[9] Pham L. H., Nguyen H. T., Van Tran C., Nguyen H. M., Nguyen T. H., Tu M. B. (2017). Arsenic and other trace elements in groundwater and human urine in Ha Nam province, the Northern Vietnam: contamination characteristics and risk assessment. *Environmental Geochemistry and Health*.

[10] Agusa T., Kunito T., Kubota R., Monirith I., Tanabe S., Tana T. S. (2002). Arsenic pollution in Cambodia. *Biomedical Research on Trace Elements*.

[11] Agusa T., Kunito T., Nakashima E. (2003). Preliminary studies on trace element contamination in dumping sites of municipal wastes in India and Vietnam. *Journal de Physique IV (Proceedings)*.

[12] Nguyen M. H., Pham T. D., Nguyen T. L. (2018). Speciation analysis of arsenic compounds by HPLC-ICP-MS: application for human serum and urine. *Journal of Analytical Methods in Chemistry*.

[13] World Health Organization (1998). *Guidelines for Drinking Water Quality*.

[14] Phuong T. D., Kokot S., Chuong P. V., Tong Khiem D. (1999). Elemental content of Vietnamese ricePart 1. Sampling, analysis and comparison with previous studies. *The Analyst*.

[15] Agusa T., Kunito T., Fujihara J. (2006). Contamination by arsenic and other trace elements in tube-well water and its risk assessment to humans in Hanoi, Vietnam. *Environmental Pollution*.

[16] Arnold H. L., Odom R. B., James W. D. (1990). *Andrew’s Diseases of the Skin: Clinical Dermatology*.

[17] Kondakis X. G., Makris N., Leotsinidis M., Prinou M., Papapetropoulos T. (1989). Possible health effects of high manganese concentration in drinking water. *Archives of Environmental Health: An International Journal*.

[18] Woolf A., Wright R., Amarasiriwardena C., Bellinger D. (2002). A child with chronic manganese exposure from drinking water. *Environmental Health Perspectives*.

[19] Hall M., Chen Y., Ahsan H. (2006). Blood arsenic as a biomarker of arsenic exposure: results from a prospective study. *Toxicology*.

[20] Agusa T., Kunito T., Minh T. B. (2009). Relationship of urinary arsenic metabolites to intake estimates in residents of the Red River Delta, Vietnam. *Environmental Pollution*.

[21] Karadas S., Sayın R., Aslan M. (2014). Serum levels of trace elements and heavy metals in patients with acute hemorrhagic stroke. *The Journal of Membrane Biology*.

[22] Ministry of Health (MOH) (2010). *National Institute of Nutrition Vietnam. General Nutrition Survey 2009–2010*.

[23] World Health Organization (WHO) (2010). *Exposure to Arsenic: A Major Public Health Concern*.

[24] Kile M. L., Houseman E. A., Breton C. V. (2007). Dietary arsenic exposure in Bangladesh. *Environmental Health Perspectives*.

[25] Diaz O. P., Leyton I., Munoz O. Contribution of water, bread, and vegetables (raw and cooked) to dietary intake of inorganic arsenic in a rural village of Northern Chile. *The Journal of Agricultural and Food Chemistry*.

[26] Del Razo L. M., Garcia-Vargas G. G., Garcia-Salcedo J. (2002). Arsenic levels in cooked food and assessment of adult dietary intake of arsenic in the Region Lagunera, Mexico. *Food and Chemical Toxicology*.

